# LncRNA HAGLR regulates gastric cancer progression by regulating the miR-20a-5p/E2F1 axis

**DOI:** 10.18632/aging.206039

**Published:** 2024-08-21

**Authors:** Qingwei Liu, Yong Li, Bibo Tan, Qun Zhao, Liqiao Fan, Zhidong Zhang, Dong Wang, Xuefeng Zhao, Yu Liu, Wenbo Liu

**Affiliations:** 1 The Third Department of Surgery, The Fourth Hospital of Hebei Medical University, Shijiazhuang, China

**Keywords:** gastric cancer, lncRNA HAGLR, miR-20a-5p, E2F1

## Abstract

Background: Gastric cancer (GC) stands as a prevalent and challenging malignancy within the gastrointestinal tract. The potential of long non-coding RNAs (lncRNAs) as biomarkers and therapeutic targets in oncology has garnered immense research interest. This study aims to elucidate the relevance, biological roles, and mechanistic pathways of LncRNA HAGLR in the context of GC.

Methods: The assessments of cell proliferation, migration, and invasion were executed using CCK-8, wound healing, and Transwell assays. The interactions between HAGLR, miR-20a-5p, and E2F1 were appraised through luciferase reporter assays, fluorescence *in situ* hybridization (FISH), and RNA immunoprecipitation (RIP). A tumor xenograft model provided *in vivo* validation for *in vitro* findings.

Results: Elevated levels of HAGLR in GC cells and tissue specimens were linked to worse patient outcomes. The inhibition of HAGLR led to a decrease in GC cell proliferation, migration, and invasion, whereas its activation prompted contrary effects. The impact of HAGLR on cell migration and invasion was notably associated with epithelial-mesenchymal transition (EMT). Through bioinformatics, luciferase reporter assays, FISH, RIP, and Western blot analyses, it was revealed that HAGLR acts as a molecular sponge for miR-20a-5p, consequently augmenting E2F1 levels.

Conclusions: The data suggest that the HAGLR/miR-20a-5p/E2F1 regulatory cascade is implicated in GC pathogenesis, offering a novel therapeutic avenue for GC management.

## INTRODUCTION

Gastric cancer exhibits a notably high occurrence in countries across Asia, including China, Japan, and South Korea. About half of all gastric cancer cases in China present at an advanced stage, posing a significant risk to health [1, 2]. The main approach for treating this disease is through surgical removal of the affected tissue. However, even with advancements in treatment options like chemotherapy and radiation therapy, outcomes for those with advanced-stage gastric cancer remain poor and the likelihood of cancer returning after surgery is still considerable [3, 4]. Consequently, it is critical to explore the molecular underpinnings of gastric cancer to identify new treatment targets.

Long noncoding RNAs (lncRNAs), which span more than 200 nucleotides, are a group of non-protein-coding gene sequences found ubiquitously across the genome [5, 6]. Their aberrant expression is a known contributor to the progression of diverse tumor types, and this includes gastric malignancies [7–9]. These lncRNAs exert their influence on cellular functions by way of epigenetic, transcriptional, or post-transcriptional regulatory means [10–11]. They have the capacity to act either as oncogenes or tumor suppressors, and in doing so, they modulate cancer cell proliferation, spread, and resistance to drugs [12]. Moreover, certain non-coding RNAs (ncRNAs)—like lncRNAs, pseudogenes, and circular RNAs (circRNAs)—serve as competing endogenous RNAs (ceRNAs). They regulate microRNAs (miRNAs) by binding to the same target sites in a competitive manner, which in turn affects gene expression [13–16]. Notably, recent findings have pointed to aberrant levels of the lncRNA HAGLR in different cancers [17, 18], but its specific role in the progression of gastric cancer requires further research. HAGLR thus represents a potential avenue for the advancement of diagnostic and therapeutic strategies in the context of gastric cancer.

The present study associates increased levels of HAGLR in gastric cancer tissue with a worsened patient outlook. In terms of function, both *in vitro* and *in vivo* tests show HAGLR’s role in promoting the growth, invasion, and spread of gastric cancer cells. Mechanistic insights suggest that HAGLR might act as a ceRNA for miR-20a-5p, leading to upregulated expression of E2F1. This study sheds light on the intricate HAGLR/miR-20a-5p/E2F1 ceRNA network active in gastric cancer, suggesting a fresh avenue for therapeutic intervention that may enhance patient outcomes.

## RESULTS

### HAGLR is upregulated in gastric cancer tissues and is associated with poor patient prognosis

**Figure 1:**
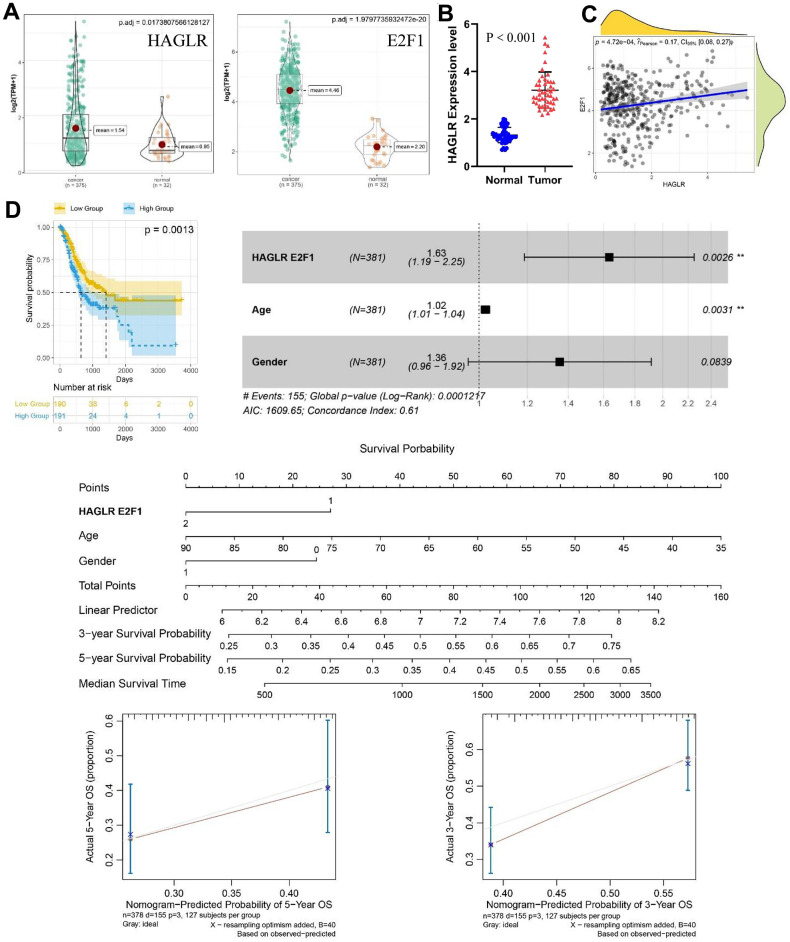
**The expression of HAGLR-E2F1 pair in GC tissues and cells and its prognostic value.** (**A**) The expression of HAGLR and E2F1 was higher in GC tissues in the TCGA database. (**B**) HAGLR was upregulated in clinical GC tissues. (**C**) Correlations of the expression of HAGLR and E2F1. (**D**) Survival analysis of HAGLR in the TCGA database.

Differential expression analysis of TCGA gastric cancer data revealed that a pair of important ceRNAs, HAGLR-E2F1, were highly expressed in cancer tissue (Figure 1A) and significantly correlated with E2F1 expression (Figure 1C). Thereafter, the expression of HAGLR in gastric cancer tissues was determined using RT-PCR. Based on the results, HAGLR expression was significantly upregulated in gastric cancer tissues compared to that in normal tissues (Figure 1B). On the basis of multivariate Cox regression analysis, the analysis also revealed a negative correlation between HAGLR levels and overall survival of gastric cancer patients. The nomogram is constructed to estimate the survival probability of three and five years, and the correction curve is drawn, which has good prediction accuracy (Figure 1D). These findings suggest that HAGLR is highly expressed in gastric cancer tissues and is associated with poor prognosis.

### Effect of HAGLR on gastric cancer cell proliferation and cell cycle

**Figure 2:**
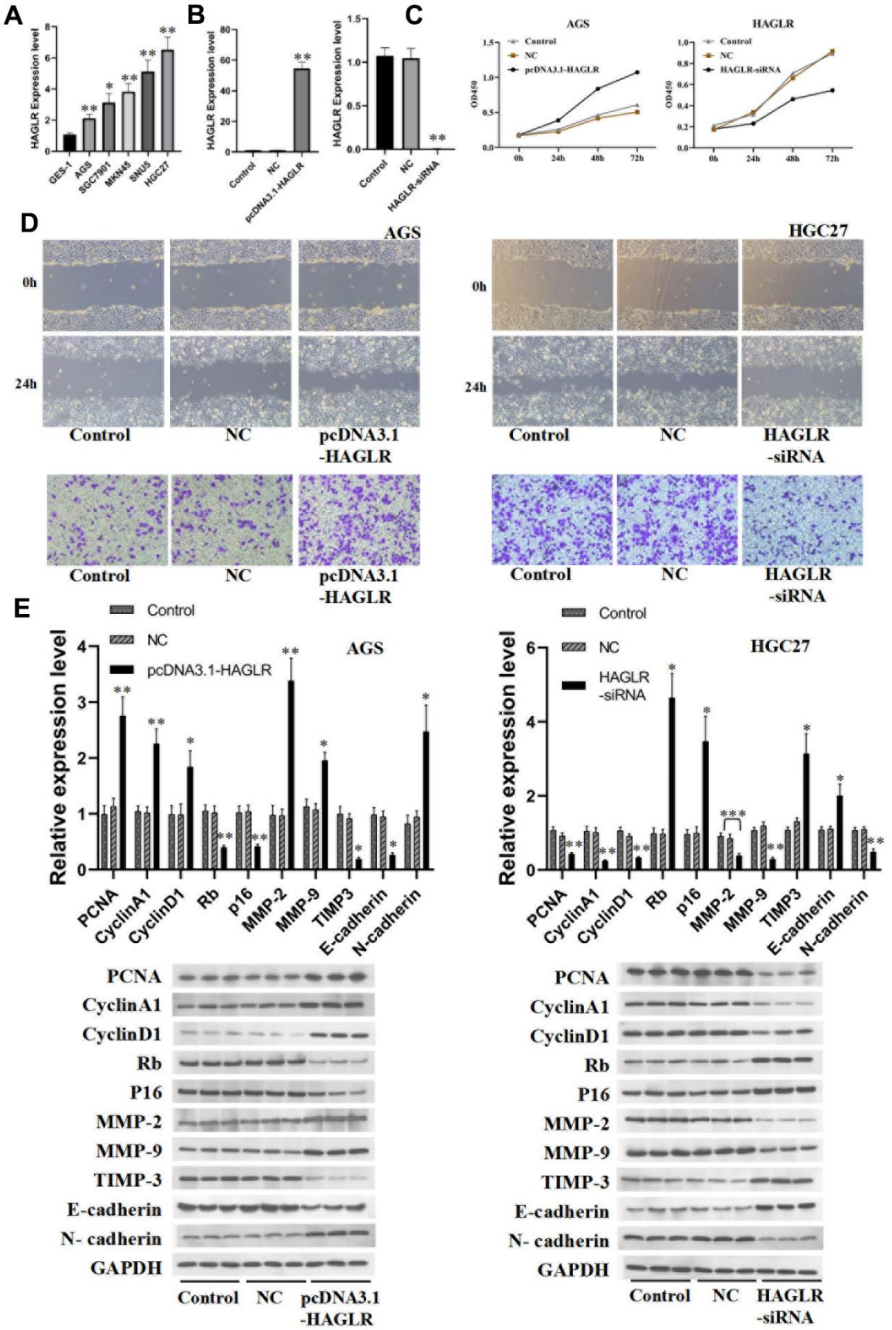
**HAGLR induces the proliferation, migration, and invasion of GC cells.** (**A**) The expression of HAGLR in various cell lines. (**B**) Knockdown or overexpressed HAGLR in AGS cell line. (**C**) The effects of differentially expressed HAGLR on cell proliferation were confirmed by CCK-8 assay. (**D**) Scratch wound healing and Transwell assays revealed the migration and invasion ability of GC cells transfected HAGLR-siRNA or pcDNA3.1-HAGLR. (**E**) Western blotting assay indicated the level of proteins with HAGLR knockdown or overexpression. (*P < 0.05, **P < 0.01, ***P < 0.001).

RT-PCR determined the levels of HAGLR in several gastric cancer cell lines, including AGS, MKN45, SGC7901, SUN5, and HGC27. HAGLR appeared to be markedly upregulated in these cells when compared to non-cancerous GES-1 cells (Figure 2A). AGS and HGC27 cells were singled out for further experimentation. Upon transfecting these cells to modulate HAGLR levels, subsequent qRT-PCR confirmed the success of the intervention (Figure 2B). Proliferation assays suggested that downregulating HAGLR hinders proliferation in HGC27 cells, while its upregulation did the same in AGS cells (Figure 2C). Western blot analysis provided insights into the proliferation and cell cycle dynamics, revealing differential effects of HAGLR manipulation on proteins like PCNA, Cyclin A1, Cyclin D1, Rb, and p16 between the cell types (Figure 2E). This indicates HAGLR’s involvement in promoting proliferation and potentially causing cell cycle arrest in gastric cancer cells.

### Effect of HAGLR on gastric cancer cell migration and invasion

Additionally, the influence of HAGLR on gastric cancer cell migration and invasion was evaluated through scratch wound healing assays, Transwell assays, and Western blotting. HAGLR silencing reduced the migratory capabilities of HGC27 cells, while enhancing those in AGS cells; outcomes were consistent across assays (Figure 2D). Examination of EMT and associated proteins by Western blot showed lowered N-cadherin, MMP-2, and MMP-9 and heightened E-cadherin and TIMP3 in HAGLR silenced HGC27 cells, with AGS displaying reverse manifestations (Figure 2E). These results align with HAGLR fostering gastric cancer cells’ migratory and invasive behaviors.

### Knockdown of *HAGLR* inhibits gastric cancer cell proliferation *in vivo*

**Figure 3:**
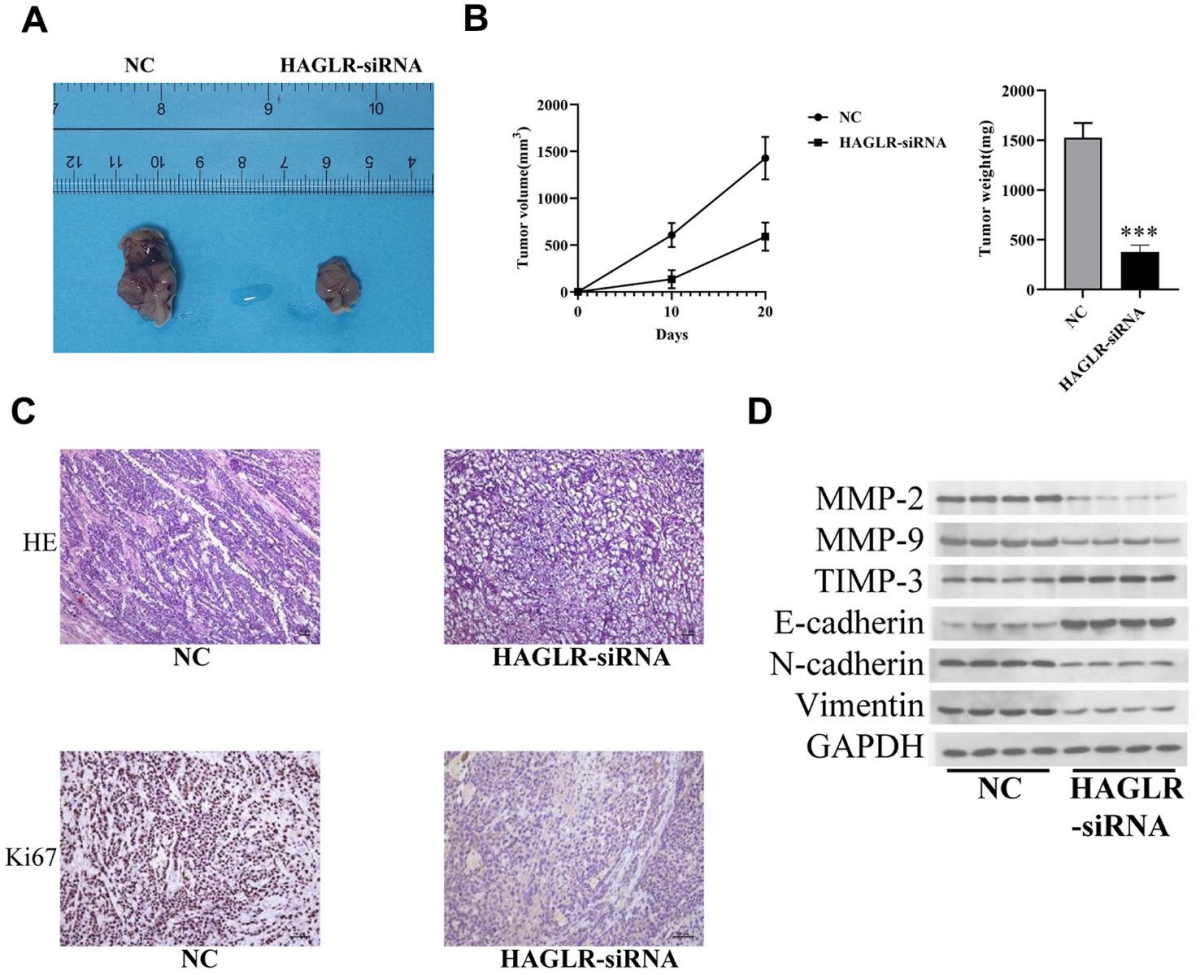
**HAGLR facilitates the tumor progression *in vivo*.** (**A**) Diagram of tumor-bearing experimental results in nude mice. (**B**) Tumor volume and weight changes in the 2 groups. (**C**) IHC analysis of the Ki-67 level. (**D**) Western blotting assay showed the levels of related proteins. (*P < 0.05, **P < 0.01, ***P < 0.001).

To assess HAGLR’s impact on tumor growth *in vivo*, we established a mouse model by injecting female nude mice with HGC27 cells that either contained shRNA targeting HAGLR or a control sequence. The mice injected with the shRNA-expressing HGC27 cells developed markedly smaller tumors, both in terms of size and weight, as compared to the control group (Figure 3A, 3B), highlighting HAGLR’s role in promoting tumor growth. Further histological analysis with H&E staining supported the inhibitory effect of HAGLR knockdown on tumor formation, and immunohistochemical analysis confirmed decreased Ki-67 proliferation marker levels in the HAGLR-suppressed tumors (Figure 3C). Western blot analysis verified that downregulation of HAGLR correlated with lower expression of proteins related to EMT and matrix metalloproteinases, including N-cadherin, vimentin, MMP-2, and MMP-9, and a concomitant increase in E-cadherin and TIMP3 levels (Figure 3D).

### HAGLR competitively binds miR-20a-5p

**Figure 4:**
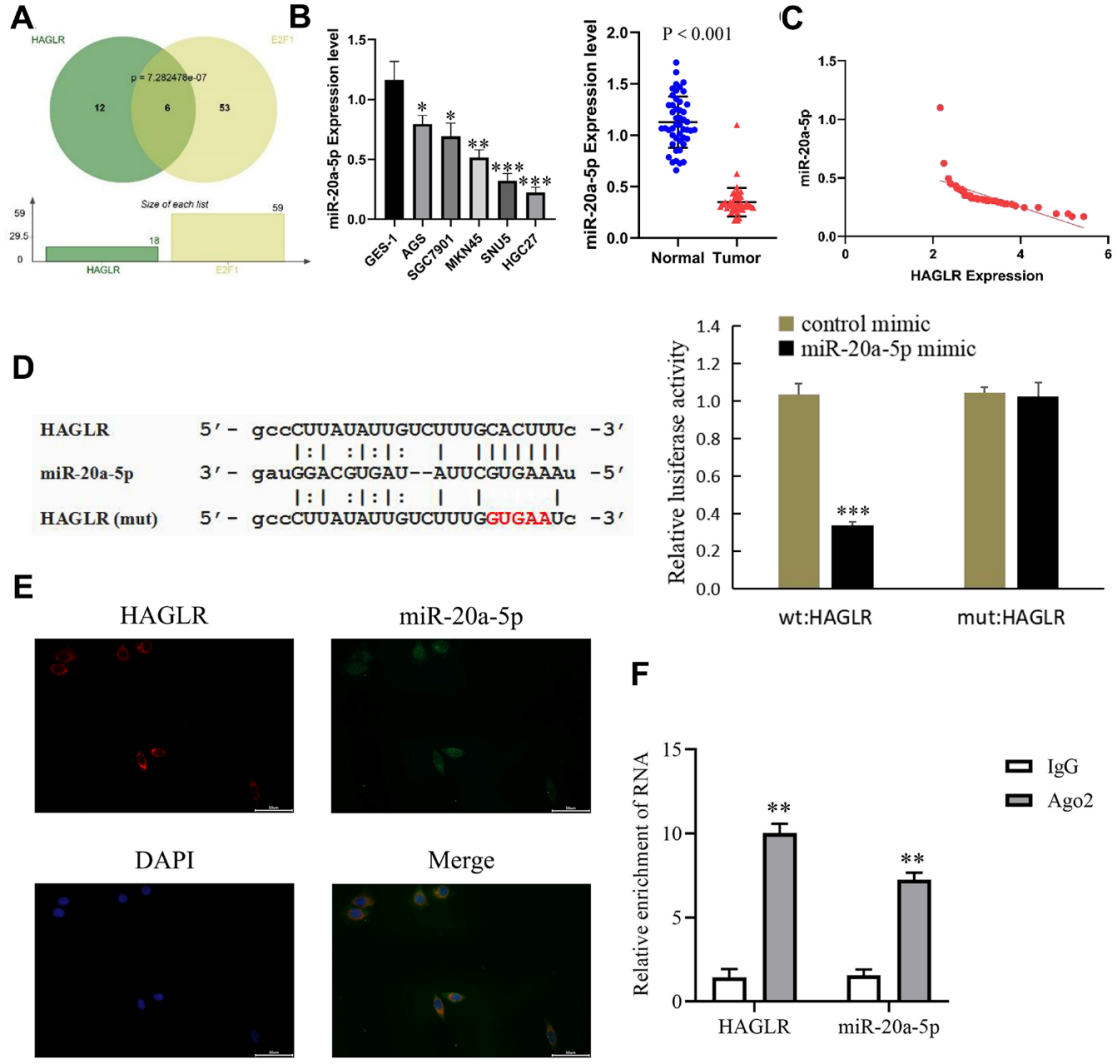
**HAGLR binds to miR-20a-5p.** (**A**) Hypergeometric test of HAGLR and the target miRNAs of E2F1. (**B**) The expression of miR-20a-5p in GC tissues and cells. (**C**) Correlations of the expression of HAGLR and miR-20a-5p. (**D**) Dual luciferase assay showed that HAGLR downregulated miR-20a-5p. (**E**) Fluorescence *in situ* hybridization results. (**F**) Diagram of RIP experimental results. (*P < 0.05, **P < 0.01, ***P < 0.001).

Utilizing lncATLAS for subcellular localization prediction, HAGLR was primarily found in the cytoplasm, suggesting a possible interaction with miRNAs. Target miRNAs of HAGLR and E2F1 were identified using a Hypergeometric test (Figure 4A), with specific focus on miR-20a-5p, implicated as an interacting miRNA through miRDB databases [19, 20]. Gastric cancer tissue analysis via qRT-PCR indicated a reduction in miR-20a-5p, implicating its tumor suppressor role (Figure 4B), and its negative correlation with HAGLR (Figure 4C). Dual-luciferase reporter assays provided evidence that HAGLR can bind to miR-20a-5p, resulting in reduced luciferase activity (Figure 4D). Fluorescence *in situ* hybridization and RNA Immunoprecipitation (RIP) experiments corroborated co-expression and increased Ago2-enrichment for HAGLR and miR-20a-5p compared to IgG (Figures 4E, 4F), verifying the sponging effect of HAGLR on miR-20a-5p.

### E2F1 regulation in gastric cancer cells is mediated by miR-20a-5p

**Figure 5:**
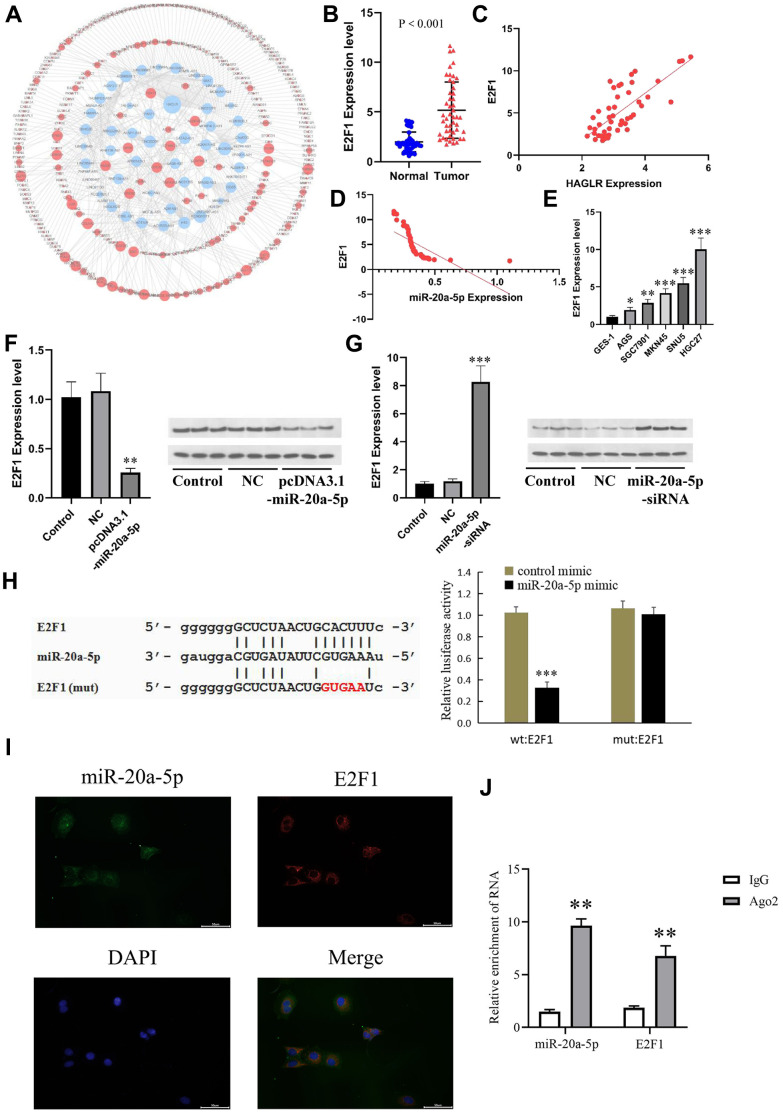
**E2F1 is the downstream gene of miR-20a-5p.** (**A**) The downstream targets of miR-20a-5p predicted by the miRDB and TargetScan databases. (**B**) The expression of E2F1 in GC tissues. (**C**) Correlations of the expression of HAGLR and E2F1. (**D**) Correlations of the expression of E2F1and miR-20a-5p. (**E**) The expression of miR-20a-5p in different cell lines. (**F**) The influence of miR-20a-5p overexpression on E2F1 mRNA and protein. (**G**) The influence of miR-20a-5p knockdown on E2F1 mRNA and protein. (**H**) Dual luciferase assay showed that miR-20a-5p downregulated E2F1. (**I**) Fluorescence *in situ* hybridization results. (**J**) Diagram of RIP experimental results. (*P < 0.05, **P < 0.01, ***P < 0.001).

Investigations utilizing miRDB and TargetScan database searches identified E2F1 as a putative target of miR-20a-5p (Figure 5A). Elevated E2F1 levels, suggested by qRT-PCR from both gastric cancer tissues and cell lines, pointed to E2F1’s possible oncogenic function (Figures 5B, 5E). Furthermore, qRT-PCR assays indicated a positive correlation between HAGLR and E2F1 (Figure 5C), and a negative relationship between miR-20a-5p and E2F1 (Figure 5D). Subsequent experiments in HGC27 and AGS cells demonstrated that miR-20a-5p modulates E2F1 expression inversely—its increase led to decreased E2F1, and vice versa (Figures 5F, 5G). Dual-luciferase assays further affirmed this interaction, with miR-20a- 5p reducing luciferase signals from an E2F1 reporter construct (Figure 5H). Fluorescence *in situ* hybridization and RIP experiments mutually showed co-expression and enrichment of Ago2-bounded miR-20a-5p and E2F1 (Figures 5I, 5J), elucidating the complex dynamics whereby E2F1’s oncogenic activity is partially mediated by miR-20a-5p.

### HAGLR regulates gastric cancer cell proliferation, migration, and invasion via negatively regulating miR-20a-5p

**Figure 6:**
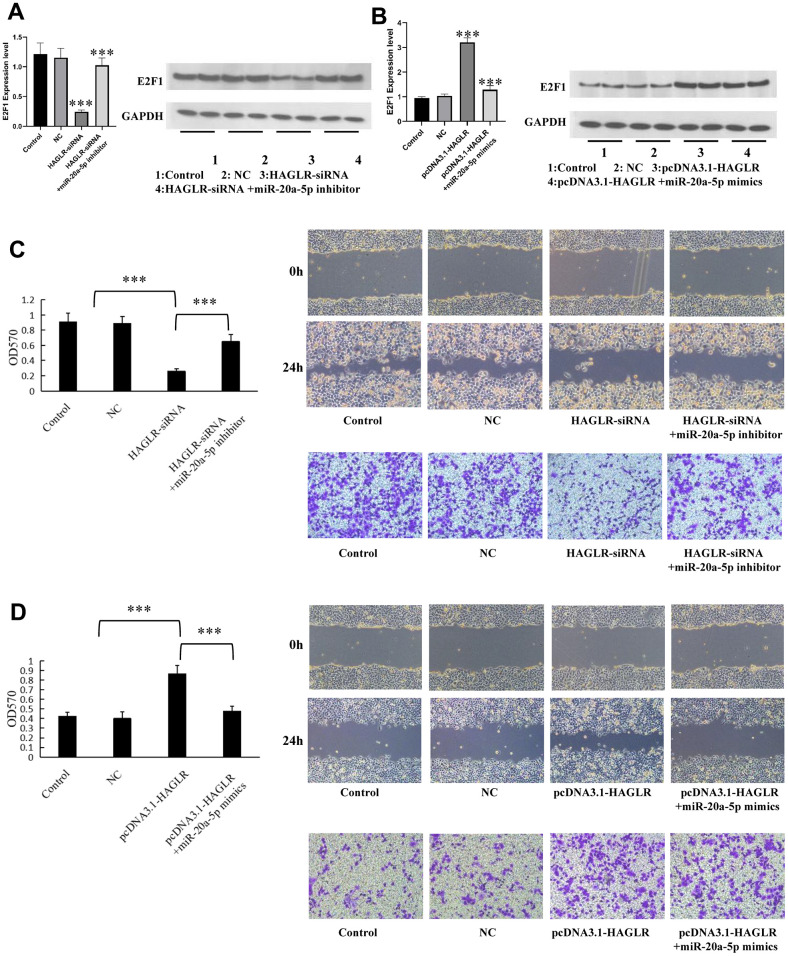
**HAGLR/miR-20a-5p axis modulates the proliferation, migration, and invasion in GC.** (**A**) HAGLR knockdown regulates E2F1 mRNA and protein. (**B**) HAGLR overexpression regulates E2F1 mRNA and protein. (**C**) HAGLR knockdown mediates the proliferation, migration, and invasion of GC cells. (**D**) HAGLR overexpression mediates the proliferation, migration, and invasion of GC cells. (*P < 0.05, **P < 0.01, ***P < 0.001).

To investigate HAGLR’s mechanism in gastric cancer, specifically its interaction with miR-20a-5p, gastric cancer cells were exposed to miR-20a-5p mimics or inhibitors to modulate HAGLR expression. When HGC27 cells were co-transfected with miR-20a-5p inhibitor, there was a partial restoration of E2F1 expression, which had been decreased by suppressing HAGLR (Figure 6A); AGS cells displayed the inverse effect (Figure 6B). These findings indicate HAGLR’s role in modulating E2F1 levels through miR-20a-5p binding. Functional assays including CCK-8, scratch wound healing, and Transwell, demonstrated that the effects of HAGLR suppression—reduced proliferation, migration, and invasion in HGC27 cells—were partially reversed through miR-20a-5p inhibition (Figure 6C). Conversely, HAGLR overexpression in AGS cells, which enhanced proliferation, migration, and invasion, was partially negated by treatment with miR-20a-5p mimics (Figure 6D). These results support the conclusion that HAGLR controls gastric cancer cell behaviors by downregulating miR-20a-5p.

## DISCUSSION

Gastric cancer remains a significant health concern worldwide, with it being a major contributor to cancer mortality [21–23]. This necessitates advancing our knowledge on its molecular underpinnings to discover new treatment options. Non-coding RNAs (ncRNAs), including miRNAs and lncRNAs, are shown to be key players in many biological processes of cancer, despite representing a vast proportion of the human genome, with only a minor portion dedicated to protein-coding [24]. Notably, ncRNAs have emerged as promising tools for diagnosis and treatment of gastric cancer (GC) [25, 26], attracting interest particularly toward lncRNA-targeted therapies in the field [27, 28]. The lncRNA known as HAGLR, implicated in various cancers such as esophageal and lung adenocarcinoma [29–31], is currently being investigated for its role in GC proliferation and migration. Our study analyzed TCGA and GEO databases, finding HAGLR overexpressed in GC samples. Further investigations established a correlation between HAGLR expression and adverse clinical features like lymph node metastasis, larger tumors, advanced TNM stages, and poor patient prognosis in GC. HAGLR was shown experimentally to enhance different malignant properties of GC cells.

The location of lncRNAs within cells determines their biological activity [32]. For example, cytoplasmic lncRNAs can sequester miRNAs affecting mRNA stability or protein synthesis and thus participate in post-transcriptional regulatory networks [33]. These networks, known as ceRNA networks, involve intricate lncRNA-mRNA interactions [34, 35]. Various lncRNAs such as LINC00857, MIR17HG, NKX2-1-AS1, LINC01234, and FAM225A have been found to manipulate specific miRNAs to influence cancer cell behavior [36–39]. In our work, bioinformatics and dual-luciferase assays revealed that HAGLR binds to miR-20a-5p, which generally acts as a tumor suppressor [40–43]. Our findings showed low miR-20a-5p expression in GC, inversely related to HAGLR expression, suggesting HAGLR’s role in oncogenesis via miR-20a-5p suppression.

Moreover, our study identified E2F1 as a direct target of miR-20a-5p. E2F1, a pivotal transcription factor for cell cycle governance, is linked to tumor progression when aberrantly expressed [44], and has a supportive role in advancing GC, amplifying the ability of cancer cells to grow and spread [45]. We observed an increase in E2F1 levels, inversely related to miR-20a-5p but positively correlated with HAGLR, indicating miR-20a-5p’s suppressive effect on the oncogenic function of E2F1.

Collectively, HAGLR functions as an oncogenic facilitator in GC progression, mediating actions through the HAGLR/miR-20a-5p/E2F1 axis. Our research contributes to a deeper understanding of GC pathophysiology and may aid in discovering new diagnostic, prognostic, and therapeutic avenues.

## CONCLUSIONS

In summary, HAGLR acts as an oncogenic lncRNA in gastric cancer, contributing to cell malignancy via the HAGLR/miR-20a-5p/E2F1 axis. This research provides insights into the molecular pathogenesis of gastric cancer and suggests that targeting this pathway could offer novel avenues for the development of diagnostics, therapeutics, and prognostic markers in this disease.

## MATERIALS AND METHODS

### Cell lines and principal reagents

The gastric cancer cell lines, AGS, MKN45, SGC7901, SUN5, and HGC27; and the normal gastric mucosal epithelial cell line, GES-1, were provided by the Fourth Hospital of Hebei Medical University Research Institute. MTT kits were purchased from Shanghai Bioengineering Co. (China). DMEM, fetal bovine serum, and RPMI-1640 medium were purchased from Gibco (USA). Quantitative real-time polymerase chain reaction (qRT-PCR) kits, reverse transcription kits, and Lipofectamine 3000 transfection reagent were purchased from Invitrogen (USA). Negative control plasmid, HAGLR overexpression plasmid, recombinant HAGLR wild-type (HAGLR-WT) plasmid, recombinant HAGLR mutant (HAGLR-MT) plasmid, miR-NC, and miR-20a-5p mimic were purchased from Beyotime (Shanghai, China). Dual-luciferase reporter gene assay kits were purchased from Promega (USA). Bicinchoninic acid (BCA) protein assay kits were purchased from Pierce (USA).

### Clinical samples

Gastric cancer tissue samples and adjacent non-tumor tissues were collected from 50 gastric cancer patients at the Fourth Hospital of Hebei Medical University. These patients had undergone gastrectomy but had not undergone relevant surgery or radiotherapy prior to operation. Immediately after resection, the tissues were frozen in liquid nitrogen and stored at -80° C. The study was approved by the Institutional Review Board of the Fourth Hospital of Hebei Medical University. All patients provided written informed consent. Complete clinical and follow-up data were obtained for all patients.

### Bioinformatics analysis

The gastric cancer dataset was generated using TCGA database (http://cancergenome.nih.gov/). Gene expression data were obtained from TCGA database using the “TCGAbiolinks” R package, which was developed specifically for integrated analysis with GDC data. Based on the original read count expression profiles, differentially expressed genes were identified using the DESeq2 R package, and genes with corrected *p*-values less than 0.05 and expression changes greater than 1-fold were identified as differentially expressed genes. For each pair of differentially expressed lncRNAs and mRNAs, the target miRNAs of known lncRNAs and mRNAs in the database were separately extracted, and the hypergeometric test was used to determine whether the miRNAs of the lncRNA-mRNA pairs had significant overlap. The “survival” and “survminer” R packages were used for survival analysis.

### Cell culture and transfection

Cells were grown in RPMI-1640 medium supplemented with 10% fetal bovine serum and maintained at 37° C under a 5% CO2 atmosphere in a cell culture incubator. Logarithmically growing cells were seeded into 6-well plates. For transient transfection, Lipofectamine 2000 was employed to introduce either the negative control plasmids or the HAGLR overexpression plasmids into the cells, which were dubbed as the negative control and HAGLR groups. After 5 hours post-transfection, the culture medium was exchanged for a fresh supply.

### qRT-PCR

After cells in the logarithmic growth phase were collected, total RNA was extracted using TRIzol and reverse transcribed into cDNA. The reaction system and conditions were based on the manufacturer’s instructions. The following qRT-PCR cycling program was employed: pre-denaturation at 95° C for 3 min, denaturation at 95° C for 20 s, annealing at 60° C for 25 s, and extension at 72° C for 25 s for 40 cycles. The following primers were employed:

HAGLR upstream primer: 5’-CTGTGACTGTCCCTTGAATACTGA-3’, downstream primer: 5’-TGTTCCTCTGCTTGACCTGG-3’;

miR-20a-5p upstream primer: 5’-TAAAGTGCTTATAGTGCAGGTAG-3’, downstream primer: 5’-GTCGTATCCAGTGCAGGGT-3’;

E2F1 upstream primer: 5’-GACGGTGAGAGCACTTCTGT-3’, downstream primer: 5’-TCAAGGGTAGAGGGAGTTGG-3’;

U6 upstream primer: 5’-CTCGCTTCGGCAGCACA-3’, downstream primer: 5’-AACGCTTCACGAATTTGCGT-3’;

GAPDH upstream primer: 5’-GACAGTCAGCCGCATCTTCT-3’, downstream primer: 5’-GCGCCCAATACGACCAAATC-3’.

GAPDH was used as the internal reference gene for HAGLR while U6 was used as the internal reference gene for miR-93-5p. Relative expression was calculated using the 2^-ΔΔCt^ method.

### CCK-8 assay

Gastric cancer cells in the active division phase were placed into 96-well plates at 5 × 10^3 cells/mL, 200 μL per well. Cell proliferation was monitored at time intervals of 0, 24, 48, and 72 h. CCK-8 reagent, 10 μL, was added to each well and cells were incubated for 4 h before the absorbance at 450 nm (A450) was measured with a spectrophotometer to create a cell growth chart.

### Scratch wound healing assay

Gastric cancer cells were prepared at 2 × 10^5 cells/mL and distributed into 6-well plates, 2 mL per well. A 10 μL pipette tip was used to create a straight scratch once the cellular layer hit 95% confluence. Plates were cleansed with serum-free DMEM before replenishing with 2 mL of the same medium. Initial scratch width (K1) was recorded with an inverted microscope. This step was followed by a further 24 hours of incubation, after which the final scratch width (K2) was measured. Scratch closure rate was calculated with the equation (K1 - K2) / K1 × 100%.

### Transwell assay

Cells, 48 hours post-transfection, were suspended in serum-free culture medium at a concentration of 2 × 10^5 cells/mL. In preparation for the assay, 24-well plates received chambers pre-coated with Matrigel. Subsequently, each Transwell chamber’s upper compartment received 200 μL of the cell suspension while the lower compartment was filled with 500 μL of medium with 10% fetal bovine serum. After a 48-hour period, cells were fixed with paraformaldehyde, crystal violet-stained, and the migrating cells counted under a microscope.

### Bioinformatics software prediction and dual-luciferase reporter gene assay

The miRDB online database helped identify potential HAGLR target genes. A recognized binding site indicated that miR-20a-5p could be a target of HAGLR. Gastric cancer cells grown to the logarithmic phase were seeded into 96-well plates and co-transfected with HAGLR-WT or HAGLR-MT plasmid along with either miR-NC or miR-20a-5p mimic. Post 48-hour incubation, firefly and Renilla luciferase activities were measured using a dual-luciferase reporter gene assay kit, thus facilitating the calculation of relative luciferase activity for each well, normalized to an internal reference.

### Western blotting

Cell lysis in gastric cancer samples was performed with RIPA buffer, followed by protein extraction and quantification via BCA protein analysis. Proteins underwent SDS-PAGE, then were transferred to a nitrocellulose membrane, blocked with skim milk, and incubated overnight at 4° C with various primary antibodies (anti-PCNA dilution ratio 1:1000, anti-CyclinA1 dilution ratio 1:1000, anti-CyclinD1 dilution ratio 1:1000, anti-Rb dilution ratio 1:1000, anti-p16 dilution ratio 1:1000, anti-MMP-2 dilution ratio 1:1000, anti-MMP-9 dilution ratio 1:1000, anti-TIMP-3 dilution ratio 1:1000, anti-E-cadherin dilution ratio 1:1000, anti-N-cadherin dilution ratio 1:1000). After washing, the membrane was exposed to horseradish peroxidase-conjugated secondary antibody at a 1:10,000 dilution and incubated at 37° C for 2 hours. The detected signals were visualized with ECL and band intensities quantified utilizing ImageJ software.

### Establishment of a tumor transplantation model in nude mice

All experiments were approved by the Institutional Animal Care and Use Committee of Hebei Medical University and conducted in strict compliance with the National Institutes of Health Guide for the Care and Use of Laboratory Animals. A 1 × 10^7^ cells/mL single-cell suspension of GC cells was prepared and injected into the axilla of one side of nude mice (aged 4-5 weeks). Mice were randomly divided into two groups. Tumors were measured once per week and tumor volumes were calculated as tumor volume (cm^3^) = 0.5 × (minimum surface diameter)^2^ × maximum surface diameter. Mice were killed 30 days after treatment, and the tumors were excised and weighed. Transplanted tumor tissues were stained with H&E and IHC, and protein expression levels were detected via Western blot.

### Immunohistochemistry

The research received approval from Hebei Medical University’s Institutional Animal Care and Use Committee and complied with NIH standards for animal care. GC cell suspensions (1 × 107 cells/mL) were injected into 4–5-week-old nude mice. The mice were divided into two groups and tumors were measured weekly. Tumor size was determined using the formula 0.5 × (smaller diameter)2 × (larger diameter). After 30 days post-treatment, the mice were euthanized, tumors were collected and weighed, then processed for histological and protein expression analysis.

### Fluorescence *in situ* hybridization assay

Prepare cell sections and perform three PBS washes for 5 minutes each. Then fix the cells using 4% paraformaldehyde for 5-10 minutes at 4° C and follow with a PBS rinse. Proceed by treating the cells with 0.5% TritonX-100 for 20 minutes, and subsequently rinse with PBS for another 10 minutes. Apply anhydrous alcohol, allow to dry, and then add the hybridization solution. Denature the probe using a PCR instrument, place it in the culture wells, and allow attachment. Incubate overnight at 37° C, wash, and block with 3% BSA. Then the digoxigenin-labeled fluorescent secondary antibody is applied for 1 hour, and cells are stained with DAPI for 10 minutes. Perform a final PBS wash for 15 minutes, remove any remaining PBS with distilled water, and air dry at room temperature, keeping the cells shielded from light. Seal the samples and examine them under a fluorescence microscope, and document the observation using an Olympus CKX53 microscope.

### RNA immunoprecipitation (RIP) assay

RIP assay was performed using the EZ-Magna RIP kit, incubating cell lysates with magnetic beads conjugated to Anti-IgG or Anti-Ago2. RNA associated with HAGLR and miR-20a-5p or with miR-20a-5p and E2F1 was quantified by qRT-PCR.

### Statistics and data analyses

Prism 9.0 was used for student t-test and analysis of variance, and results are presented as means with standard deviations. Linear regression was used to explore the relationship between two genes, while Fisher's exact test examined associations with clinical features in GC. Survival analysis utilized Cox models and Kaplan–Meier curves, with a p-value less than 0.05 indicating significance.

### Data availability statement

The corresponding author can provide the raw data used and analyzed in this study if necessary.

### Consent for publication

All the authors of this review give their consent for publication.
